# Tissue-specific promoter-based reporter system for monitoring cell differentiation from iPSCs to cardiomyocytes

**DOI:** 10.1038/s41598-020-58050-2

**Published:** 2020-02-05

**Authors:** Katarzyna Fiedorowicz, Natalia Rozwadowska, Agnieszka Zimna, Agnieszka Malcher, Katarzyna Tutak, Izabela Szczerbal, Karolina Nowicka-Bauer, Magdalena Nowaczyk, Tomasz J. Kolanowski, Wojciech Łabędź, Łukasz Kubaszewski, Maciej Kurpisz

**Affiliations:** 1 Institute of Human Genetics, Polish Academy of Sciences, Poznan, Poland; 2 Department of Genetics and Animal Breeding, Poznan University of Life Sciences, Poznan, Poland; 3 Department of Spondyloortopaedics and Biomechanics of the Spine, W. Dega University Hospital, Poznan University of Medical Sciences, Poznan, Poland

**Keywords:** Bioluminescence imaging, Fluorescence imaging, Stem-cell biotechnology

## Abstract

The possibility of using stem cell-derived cardiomyocytes opens a new platform for modeling cardiac cell differentiation and disease or the development of new drugs. Progress in this field can be accelerated by high-throughput screening (HTS) technology combined with promoter reporter system. The goal of the study was to create and evaluate a responsive promoter reporter system that allows monitoring of iPSC differentiation towards cardiomyocytes. The lentiviral promoter reporter system was based on *troponin 2* (*TNNT2*) and *alpha cardiac actin* (*ACTC*) with firefly luciferase and mCherry, respectively. The system was evaluated in two *in vitro* models. First, system followed the differentiation of *TNNT2-luc-T2A-Puro-mCMV-GFP and hACTC-mcherry-WPRE-EF1-Neo* from transduced iPSC line towards cardiomyocytes and revealed the significant decrease in both inserts copy number during the prolonged *in vitro* cell culture (confirmed by I-FISH, ddPCR, qPCR). Second, differentiated and contracting control cardiomyocytes (obtained from control non-reporter transduced iPSCs) were subsequently transduced with *TNNT2-luc-T2A-Puro-CMV-GFP and hACTC-mcherry-WPRE-EF1-Neo* lentiviruses to observe the functionality of obtained cardiomyocytes. Our results indicated that the reporter modified cell lines can be used for HTS applications, but it is essential to monitor the stability of the reporter sequence during extended cell *in vitro* culture.

## Introduction

Heart disease has been a major cause of morbidity and mortality worldwide. Since the heart has limited potential to regenerate itself, the main treatment of failing heart has been based on interventional cardiology, pharmacological treatment and heart transplantation^1–3^. The development of *in vitro*-induced pluripotent stem cell (iPSC) technology by Yamanaka and colleagues^4^ opened a new field and novel therapeutic approaches based on the reprogramming of somatic cells^5^ and their subsequent differentiation^6,7^ have emerged. Although there have been tremendous improvements in the protocols used to obtain cardiomyocytes, with purities ranging from 80–95%^8^, there are still unresolved issues, such as their immature phenotype^9–11^, which should be improved before iPS-derived cardiomyocytes could be further considered for application in human cellular therapies. The currently available methods for somatic cell reprogramming into iPSCs to differentiate it from the next one that is somatic cells into cardiomyocytes are time consuming and may pose a potential risk for teratoma formation *in vivo*^12^. Another new, promising and intensively investigated approach to obtain cardiomyocytes is the direct reprogramming of somatic cells into cardiomyocytes^13^. This appealing technique does not require the full reprogramming of cells into iPSCs, but may involve partial reprogramming via cardiac progenitor cells. Unfortunately, so far published protocols have not been very effective^14,15^ and based on induction with transcription factors^16^ or viral transduction^17^, which makes the process expensive and not clinically applicable. Additionally, one may change the selection of small molecule combinations to replace the currently used genomic origin factors^18^.

Finding solutions to the challenges of clinical application might be accelerated through the use of high-throughput screening methods combined with promoter reporter system that provides information on the cell differentiation stage. The lentiviral vector systems have been widely used due to their efficient transduction of dividing and non-dividing cells^19^, as well as their ability to integrate large transgenes into host genomes. Unfortunately, the data on lentiviral copy number stability and its influence on reporter expression are limited.

Presented here, is a sensitive and responsive promoter-reporter system that can monitor and validate cardiomyocyte differentiation processes. We have characterized the stability of this system with the future goal of adapting it to high-throughput technologies.

The system was designed to contain two core cardiac-specific gene promotors: *troponin 2* (*TNNT2*) that regulates muscle contraction in response to changing calcium ion concentration^20^, and *alpha cardiac actin* (*ACTC*), which is the major protein of the thin filament responsible for generating and transmitting force from the sarcomere to the syncytium^21^. The system was evaluated on two levels: tracking the cell differentiation into cardiomyocytes of the *TNNT2/ACTC*-modified iPSC line at different passages (concominantly determining the insert copy number) and by *ACTC/TNNT2* lentiviral transduction of already differentiated and contracting iPSC-derived control cardiomyocytes.

## Results

**Figure 1:**
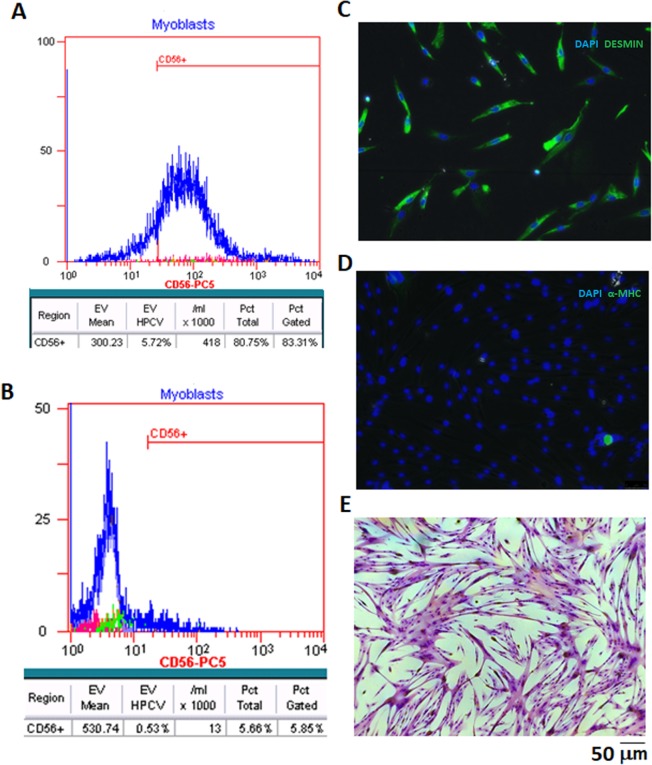
Myoblast characteristics. (**A**) Flow cytometry detected approximately 83% of CD56+ myoblasts cells in the isolated skeletal muscle population. (**B**) Isotype control (IgG1-PC5). (**C**) Immunofluorescence image of myoblasts stained with anti-desmin antibody (green) with nuclear dye DAPI (blue); (**D**) Immunofluorescence image of myoblasts stained with anti-α-MHC (myosin heavy chain) antibody (green) with nuclear dye DAPI (blue) (**E**) Multinuclear tube formation test confirmed the ability of cells to differentiate *in vitro*, scale bars = 50 µm.

The flow cytometry analysis of myoblasts, which were the initial point for iPSCs induction, revealed that approximately 83% of cells in the population were CD56+ positive (Fig. 1A). (The isotype control is presented in Fig. 1B). The marker of myoblasts (desmin) was highly expressed (Fig. 1C), while the marker of differentiated cells (MHC) was expressed at a very low level (Fig. 1D). The positive results of the myotube formation test showed that the characterized cells retained their functionality *in vitro* (Fig. 1E).

**Figure 2:**
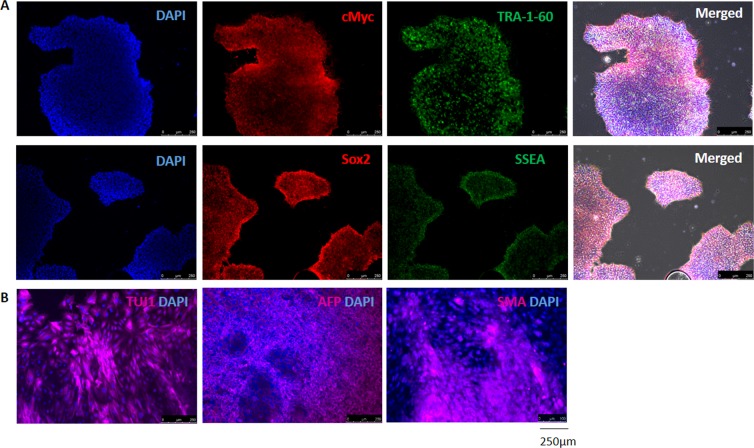
Morphology and expression of iPSC markers. (**A**) iPSCs derived from human myoblasts were expanded and characterized. Expression of undifferentiated cell markers was confirmed by immunostaining with anti-c-myc, anti-TRA, anti-Sox2 and anti-SSEA antibodies. Nuclear dye = DAPI (blue). Scale bar = 250 µm. (**B**) The *in vitro* spontaneous differentiation of iPSCs to embryoid bodies (EBs) in suspension *in vitro* culture was followed by monolayer culture. Cells of ectoderm, endoderm and mesoderm lineages were confirmed by immunostaining with neural class III, β-tubulin (TUJ-1), α-fetoprotein (AFP) and smooth muscle actin (SMA). Nuclear dye = DAPI (blue). Scale bar = 250 µm.

The iPSCs derived from previously characterized myoblasts were cultured *in vitro* for at least 15 passages, and the appropriate colony morphology was monitored under the microscope. The markers of undifferentiation were confirmed by immunofluorescent staining with anti-Sox2, anti-c-myc, anti-SSEA and anti-TRA-1-60 antibodies (Fig. 2A). Moreover, functional tests revealed that the iPSCs were able to spontaneously form embryoid bodies and differentiate into three germ layers. As shown in Fig. 2B, cells were positive for TUJ1 -III-β-tubulin (ectoderm marker), AFP- α-fetoprotein (endoderm marker) and SMA- alpha smooth muscle actin (mesoderm marker).

**Figure 3:**
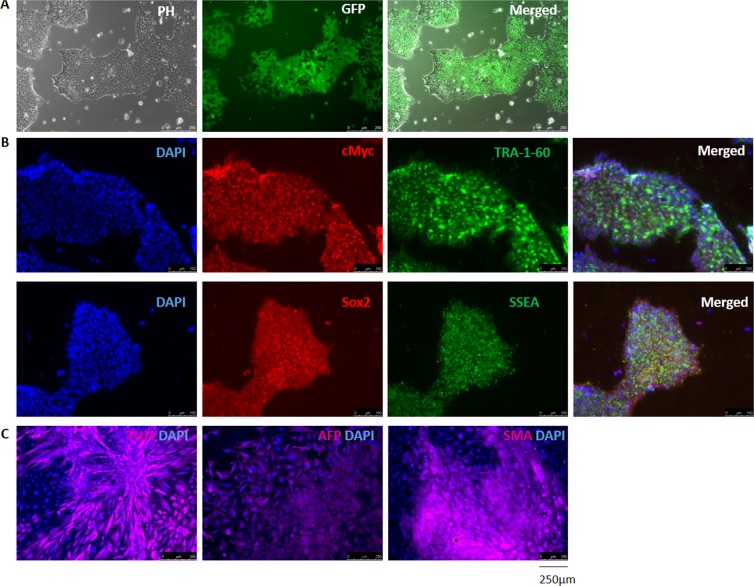
Morphology and marker expression in *TNNT2/ACTC* reporter-modified iPSC line. (**A**) The phase contrast image showed that modified *TNNT2/ACTC* iPSCs preserved their typical cell colony morphology; the GFP signal indicated the transduction efficiency. Scale bar = 250 µm. (**B**) *TNNT2/ACTC* reporter-modified iPSC line expressed classical undifferentiated cell markers: c-myc, TRA-60, Sox2 and SSEA; nuclear dye = DAPI (blue). Scale bar = 250 µm. (**C**) *TNNT2/ACTC* reporter-modified iPSC line spontaneously differentiated *in vitro* via embryoid bodies (EBs) and expressed ectoderm (neural class III β-tubulin- TUJ-1), endoderm (α-fetoprotein - AFP) and mesoderm (smooth muscle actin - SMA) markers; nuclear dye = DAPI (blue). Scale bar = 250 µm.

Next, the iPSCs were transduced with *TNNT2-luc-T2A-puro-mCMV-GFP and hACTC-mCherry-WPRE-EF1-Neo* lentiviral particles. Since GFP is controlled by a constitutive mCMV promoter, we could clone positive GFP cells to obtain a population with the *TNNT2* transgene at a purity of 94,6% (data not shown). To achieve a pure population of cells carrying the *ACTC* transgene, transduction was followed by selection with G418 for 7 days. The *TNNT2-luc-T2A-puro-mCMV-GFP/hACTC-mCherry-WPRE-EF1-Neo*-modified iPSC line (termed the TNNT2/ACTC iPSC line) preserved a typical-compact morphology with clearly defined margins (Fig. 3A). Fluorescence immunostaining experiments with anti-Sox2, anti-c-myc, anti-SSEA and anti-TRA-1-60 (Fig. 3B) confirmed the expression of undifferentiated cell markers in the generated cell line. The lentiviral reporter modification did not change the ability of cells to differentiate into three germ layers via EB formation (Fig. 3C).

**Figure 4:**
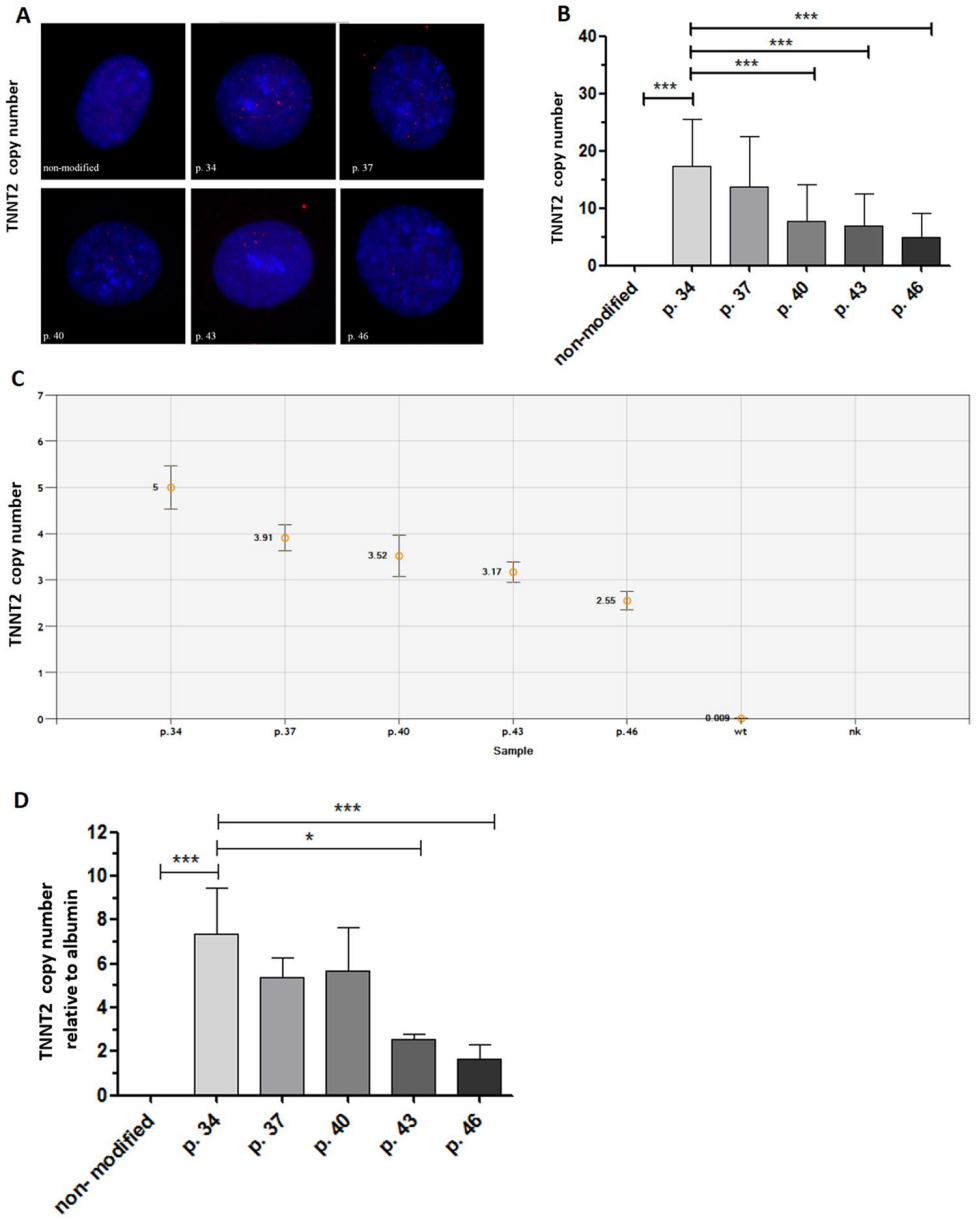
Determination of *TNNT2* copy number in *TNNT2/ACTC* reporter-modified iPSCs. (**A**) Representative images of I-FISH signals with complementary probe generated from *TNNT2* vector in the cells at passages 34, 37, 40, 43 and 46. As a negative control, reporter unmodified cells were used. Images were taken at 100x magnification. (**B**) Graphic presentation of I-FISH signals in 50 nuclei for cells at passages 34, 37, 40, 43, and 46. Unmodified cells were used as negative control. Values are given as the mean ± SD, ***p < 0.001. (**C**) *TNNT2* insert copy in modified iPSCs at cell passages 34, 37, 40, 43 and 46, and reporter-unmodified cells were evaluated by ddPCR; values are given as the mean ± SD for ddPCR triplicates, ***p < 0.001. (**D**) The reporter-modified iPSC line was expanded, and the average insert copy number in the cell population was estimated in cell passages 34, 37, 40, 43 and 46 compared to reporter-unmodified cells by qPCR relative to *Albumin*; values are given as the mean ± SD for qPCR triplicates; *p < 0.05, ***p < 0.001.

The *TNNT2/ACTC* reporter-modified iPSCs were further expanded and collected at passages 34, 37, 40, 43 and 46. To accurately determine *TNNT2* vector copy number interphase FISH (I-FISH -with complementary *TNNT2* probe generated from the vector) was performed. The identified average copy number per cell was 17 for cells at passage 34. A significant drop was observed every three passages starting from passage 40 (Fig. 4A,B), whereas cells without any inserted copy were detected in approximately 1% of the cell population for each passage (data not shown). Microscopic analysis of the I-FISH preparations showed that reporter vectors were inserted mostly as 2–3 tandem repeats, with signal usually thoroughly distributed in the cell nuclei (Fig. 4A,B). An analysis of several metaphases suggested that the vectors were inserted in the distal parts of chromosomes and were observed in both chromatids (data not shown). The *TNNT2* insert copy number was also estimated using digital droplet PCR (ddPCR) and quantitative real-time polymerase chain reaction (qPCR). The results of ddPCR also have shown a decrease in TNNT2 copy number with values ranging from 5 copies at passage 34 to 2.5 copies at passage 46 (Fig. 4C). As shown in Fig. 4D, the average copy number of *TNNT2* insert identified by *qPCR* was the highest at passage 34 (reaching 7.3 copies) and decreased significantly with the number of passages (reaching 1.6 copies in cells of passage 46). Additionally, the estimation of ACTC vector copy number was performed. In case of ACTC, I-FISH probe generated from ACTC vector (hybridized only to samples from cells of passage 40)revealed approximately 15 integrated copies per cell (Supplementary Fig. S1A). The estimation of *ACTC* inserts in the iPSC population assessed by qPCR showed that the number of copies decreased from 5.6 in cells of passage 34 to 1.3 in cells of passage 46 (Supplementary Fig. S1B).

**Figure 5:**
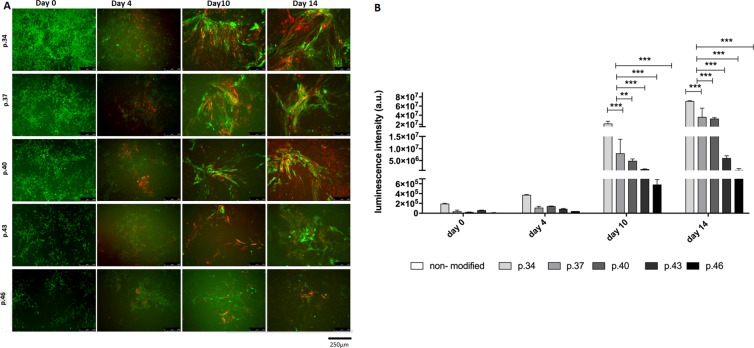
Differentiation of *TNNT2/ACTC* reporter-modified iPSCs at passages 34, 37, 40, 43, and 46 and reporter-unmodified cells towards human cardiomyocytes. (**A**) iPSCs were differentiated using a PSC protocol. Fluorescence images were taken at the beginning of differentiation (day 0), showing positive GFP signal generated by constitutively expressed mCMV promoter in the *TNNT2-luc-T2A-Puro-CMV-GFP* plasmid. Images taken at day 4 show emerging mCherry signal (passages 34–46) that became clearly visible at day 10 and increased to the end of experiment (day 14). Higher mCherry and GFP expression levels were found in cell samples at passages 34–40 than in cells at passages 43–36. Unmodified iPSCs were used as a negative control. Scale bar = 250 µm. (**B**) The level of firefly luminescence measured at day 0 and 4, 10 and 14 days after initiation of differentiation in *TNNT2/ACTC* reporter-modified cells at passages 34, 37, 40, 43, and 46. A significantly elevated luminescence signal was detected in cells at passage 34 vs. cells at passages 37, 40, 43 and 46 at day 10 and at day 14. Unmodified cells were used as a control. Measurements were performed in triplicate for each cell passage number; values are given as the mean ± SD; *p < 0.05, **p < 0.01, ***p < 0.001.

To verify if the copy number influences the sensitivity of the reporters, the cell differentiation process towards cardiomyogenic lineage of the *TNNT2/ACTC* iPSC line from cell passages 34, 37, 40, 43, and 46 was performed (reporter-unmodified cells served as a control). The GFP signal driven by the mCMV constitutive promoter (from *TNNT2-luc-T2A-Puro-mCMV-GFP*) allowed observation of how the differentiating cells aligned and how their morphology changed during the course of the experiment (Fig. 5A). Images taken at the beginning of the experiment showed that the cells of passages 43 and 46 revealed lower GFP expression levels in comparison to the other cell samples. Images taken on days 4, 10 and 14 of differentiation showed the activity of the *ACTC*-mCherry reporter to be clearly visible after 10 days and increased over time (Fig. 5A). Moreover, the expression of the mCherry reporter was more clearly visible under the microscope in the cells of passages 34–40 than in cells of passages 43 and 46 (Fig. 5A). The bioluminescent signal measured at the same time points reported the activity of TNNT2 promoter. The highest increase in firefly luciferase activity was detected for cell passage 34 at days 10 and 14 in comparison to days 0 and 4. A statistically significant decrease of luminescence in cells of passages 37, 40, 43, and 46 at days 10 and 14 was observed when compared to cells of passage 34 (Fig. 5B).

**Figure 6:**
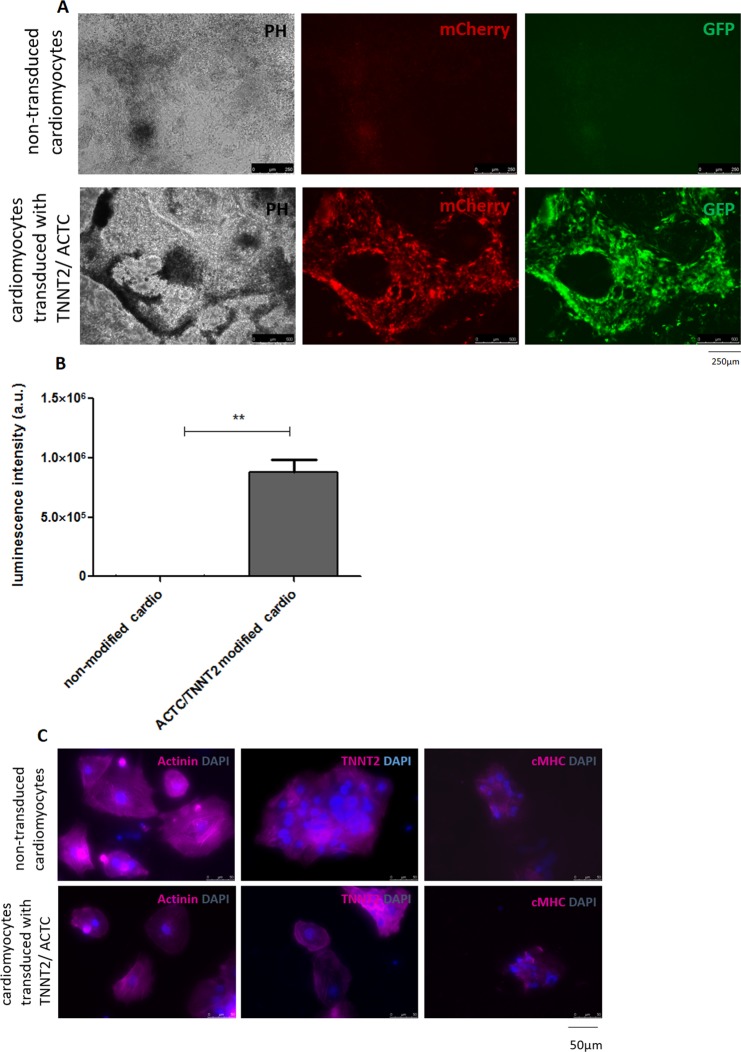
*TNNT2* and *ACTC* promoter reporter system verification in contracting human cardiomyocytes. (**A**) Reporter unmodified iPSCs were differentiated into contracting cardiomyocytes (14 d) and were then transduced with *TNNT2-luc-T2A-Puro-CMV-GFP* and *hACTC-mcherry-WPRE-EF1-Neo* lentiviruses. The figure shows corresponding images of phase-contrast, mCherry reporting actin (ACTC) expression and GFP signals generated from the constitutive mCMV promoter of the *TNNT2-luc-T2A-Puro-CMV-GFP* plasmid. Scale bar = 250 µm. (**B**) Firefly luminescence signal intensity reporting *Troponin 2 (TNNT2)* expression in *TNNT2/ACTC*-transduced cardiomyocytes measured at 72 h post transduction vs. unmodified cardiomyocytes. Values are given as the mean ± SD for the triplicates, **p < 0.01. (**C**) The immunofluorescence staining confirmed the presence of cardiomyocyte markers: ACTC, TNNT2 and cMHC, in reporter unmodified and TNNT2/ACTC reporter-modified cells. Scale bar = 50 µm.

To examine the efficiency and specificity of the reporters, we introduced them into contracting cardiomyocytes initially derived from reporter-unmodified iPSCs. Incubation with *TNNT2* and *ACTC* lentiviral vectors was carried out for 24 h. At 72 h post transduction, positive GFP and mCherry signals were observed (Fig. 6A). The bioluminescence intensity reported by *troponin 2* promoter activity was also measured at 72 h post transduction and was significantly increased in comparison to unmodified cardiomyocytes (Fig. 6B). The specificity of the reporters and the cardiac character of both (reporter-unmodified and *TNNT2/ACTC* reporter-modified) was confirmed by immunofluorescence staining for ACTC, TNNT2 and cMHC markers, as shown in Fig. 6C. We also observed that genetic modification did not disturb the contraction ability of the cells (Supplementary Data, Movie 1).

## Discussion

Currently, one of the biggest challenges in pro-regenerative heart cellular therapies is to generate large amounts of mature cardiomyocytes *in vitro*. Intensively developing platforms for disease modelling or drug testing utilize large amounts of cardiomyocytes from *in vitro* cell culture. Increasingly, the modern technologies applied in these studies are fully automated and work in a high-throughput screening mode^22^ often supplied with artificial intelligence. Thus, to be able to fully use the potential of stem cells, it is necessary to create tools that can properly and carefully verify the results of stem cell *in vitro* propagation. To this end, we have developed and validated a promoter gene reporter system that enables the visualization and quantitation of human cardiomyocytes, as well as monitoring the cell differentiation process. We based the system on two cardiac-specific gene promoters: *troponin2* (*TNNT2*) and *cardiac alpha actin* (*ACTC*). As it has been shown previously, the expression level of these biomarkers may be modulated by the cell differentiation protocols^23^. Addis *et al*.^24^ as well as Hescheler *at al*.^25^ have already proven that reporter systems based on the *troponin 2* and *cardiac alpha actin* gene can be successfully used to image cell differentiation. However, our work for the first time demonstrated that promoter reporter system needs to be carefully verified with respect to its stability of insert copy number during prolonged *in vitro* cell culture.

Importantly, for clinical application purposes, the source of the cell type used for reprogramming and differentiation seems to play a critical role^26^. Ideally, somatic cells should be easily accessible, have the potential to proliferate^27^ and, according to the so called “epigenetic memory” hypothesis, have similar embryonic origin^28^. In general, the discussion on “epigenetic memory” is controversial and extremely interesting. On one hand, there are studies by Ninshino, *et. al*. who revealed that the primary cell source may not be absolutely essential for redifferentiation^29^. On the other hand, Pianezzi *et. al*. in their recent paper showed that iPSCs from cardiac somatic cell sources differentiated into mature cardiomyocytes more efficiently compared to the cells from non-cardiac somatic cell sources^28^.

Taking into account the previous success in our group with human myoblasts^30–32^ and the limited investigations on iPSCs generated from human skeletal muscle progenitors, with the aim to subsequently differentiate them into cardiomyocytes, we have based our experimental procedure on well-characterized (Fig. 1A–E) skeletal muscle progenitor cells. As we and others^33^ have shown, myoblast-derived iPSCs exhibit typical characteristics with respect to their morphology, pluripotency markers and differentiation potential (Fig. 2A,B).

Inspired by the work of Motamedi *et al*.^23^, in which the authors used a bio- and chemi- informatics-based approach to select small molecules that contribute to iPSC differentiation, we have attempted to create and characterize a promoter-based reporter cell line with the goal of adapting it to a high-throughput screening system in the future. The high-throughput system is expected not only to automatically culture the cells but also to apply small molecule cocktails that may optimize the differentiation process. Because HTS utilizes a large amount of iPSCs, extending the *in vitro* cell culture, we first generated a pure population of *TNNT2/ACTC* reporter-containing cells with confirmed pluripotent character (as it is shown in Fig. 3A–C) and then expanded the available cell line. Cells collected from passages 34–46 were used to study the insert copy number and promoter reporter activity during subsequent differentiation.

The determination of *TNNT2* insert copy number was performed by three independent methods- I-FISH, ddPCR and qPCR. I-FISH, as a time consuming and technically challenging method, is not commonly used but it allowed the localization and distribution of insert copies in the cell nuclei (Fig. 4A,B and Supplementary Fig. S1A). One of the recent studies^ 34^ on lentiviral integration in a keranocyte genome revealed a unique pattern. Using ligation-mediated PCR followed by sequencing, it was shown that lentiviral integration site within known genes was favoured in introns over exons. Moreover, the sequences of introns did not represent junk DNA, but played a key role in gene expression and regulation. There are also strong indications that the integration site profiles may be influenced by host factors such as cell growth timing or cell cycle^35,36^. Ronen *et al*.^37^ have shown that in bone marrow cells, lentiviral vector integration sites undergo negative selection and become less frequent over prolonged *in vitro* cell culture, suggesting negative selection. Up to date, methods based on PCR techniques have emerged as a tool for determination of copy number quantification^38^. Although digital droplet PCR technique (ddPCR) possesses several advantages, such as independence of the standard curve and improved precision^39^ it is not yet easily accessible for many laboratories. In case of *TNNT2* insert copy number, the results obtained from both ddPCR and qPCR revealed a decrease over prolonged *in vitro* culturing (Fig. 4C,D). In principle ddPCR offers a more precise copy number determination, but our results showed that careful design of the qPCR assay led to a similar conclusion on inserted copy number decrease between cells at different passages as well as documented comparable data. However, values obtained from the I-FISH technique for each cell passage were much higher when compared to both PCR techniques. This is an intriguing observation, because due to the possibility of the direct visualization of inserted copies, I-FISH seems to be the exact method of copy number quantification. Therefore, the additional experiment concerning determination of *ACTC* copy number vector was performed. The estimated *ACTC* copy number by I-FISH was about 15 for cells at passage 40 revealing the same high amount of copies as for *TNNT2* in I-FISH experiments. At the same time, qPCR results showed a decrease in ACTC copy number (from 5.6 copies to 1.3 copies) as for *TNNT2* plasmid, what supported the observation that the decrease in insert copy number over prolonged cell *in vitro* culture might be a common feature of lentiviral plasmids (Supplementary Fig. S1), but not equal by presented here techniques.

In the next step, *TNNT2/ACTC* reporter-modified iPSCs from passages 34–46 were differentiated towards the cardiomyocyte lineage. The best results of promoter reporters activity during the cell differentiation procedure were obtained for cells at passages 34–40 in which elevated expression levels of *troponin2* (*TNNT2*) and *cardiac alpha actin* (*ACTC*) were clearly detected at 10 d from the initiation of cell differentiation and were still on an increase at the end of the experiment (14 d) (Fig. 5A). The decreasing copy number of TNNT2 and ACTC over prolonged *in vitro* cell culture was reflected by decreasing reporter signal intensity during cell differentiation. Similar results have been reported by Migliaccio, who cultured the K562 cell line and observed a decline in GFP expression. As Migliaccio explained, the instability during prolonged *in vitro* cell culture is probably caused by two possible integrations sites: abundant unstable and rare stable. Abundant unstable sites permit transcription, but transgenes are quickly eliminated, whereas rare stable sites allow efficient transcription and long-term integration^40^.

Problems with the detection of reporter signal intensity in cases of fluorescent reporters (such as mCherry or GFP) might also be caused by limitations in microscope resolution. For example, Cinelli and coworkers^41^ reported an inability to track GFP signal protein, and Migliaccio *et al*. showed that the minimal reporter insert copy number allowing signal detection was 3. In the case of *TNNT2/ACTC* reporter-modified iPSCs aimed for differentiation, in cells with 4 or less integrated transgene copies, as determined by qPCR, visibility of GFP and mCherry was restricted. In our view, difficulties with the microscopic observation of positive fluorescent signals during the cell differentiation process were also partially due to high background noise arising from the cell morphology. Therefore, to solve this problem and to increase the read out sensitivity, we have based the *TNNT2* reporter on bioluminescent signal.

Our promoter-based reporter system was also functionally validated by transduction of already differentiated and contracting human cardiomyocytes delivered from reporter unmodified iPSCs. Here, we could observe fluorescent mCherry and bioluminescence signal at 72 h post transduction (Fig. 6A,B). In addition, we have observed that reporter-unmodified and *TNNT2/ACTC*-modified cardiomyocytes of myogenic origin obtained from myoblasts through the iPSC stage differentiated similarly with high and similar efficiency. Both began spontaneous contraction approximately 12 d after the initiation of cell differentiation and were positive for cardiac markers (Fig. 6C). Additionally, our previously published investigations indicated that extended *in vitro* cell culture may lead to the improvement of cardiomyocyte maturation parameters, such as sarcomere organization, accumulation of cardiac mitochondrial density and transition from alpha to beta MHC isoform^42^.

### Final remarks

The future applications of iPS derived cardiomyocytes combined with high-throughput screening technologies, will greatly impact not only the understanding of genetic reprogramming and trans-(differentiation) mechanisms but also may promote novel cardiac drug discoveries or disease modelling techniques. We believe that promoter-based gene reporter systems may contribute to these investigations by successful selection of genetically reprogrammed cells from the screened cell populations. Our investigation led to the very important conclusion that to avoid losing reporter activity, it is necessary to often check the insert copy number while propagating large quantities of transduced cells for high-throughput screening purposes.

Cardiomyocytes can also be generated via the direct reprogramming of somatic cells. To date, established protocols have used fibroblasts as a source of cells^15,43,44^. However, this method is very time consuming, inefficient (1–20%), technically challenging and the obtained cells often failed to express cardiac-specific markers^15^. Therefore, the aforementioned developmental similarity of myoblasts to cardiac myocytes, together with their strong resistance to hypoxic conditions and a long-term capacity for cell proliferation^45^, make skeletal muscle-derived cells strong candidates for use in cellular therapy. In our view, cardiomyocytes originating from myoblast progenitor cells hold a great promise for regenerative medicine applications. Thus, to fully confirm their feasibility for therapy, our future investigations will focus on the functional properties of these cells in *in vivo* animal functional studies with failing heart.

## Materials and Methods

### Myoblast cell culture

Skeletal muscle stem cells were isolated from remaining tissue fragments after anterior cruciate ligament (ACL) surgical procedure. The volunteer was a 34-year-old male. Written informed consent was obtained from the study participant for the tissue donation and all the procedures, including protocols based on recommendations for human tissue collection from the Local Ethical Committee, University of Medical Sciences, Poznan. We confirm that all methods used were performed in accordance with the relevant guidelines and regulations. At the same time we should like to assure that all the experimental protocols used in this study were approved by the Local Bioethical Committee, Poznan, Poland.

Cells were propagated *in vitro* and passaged as previously described^31^. Briefly, the cells were cultured in standard Dulbecco’s modified Eagle medium with 4.5 g/l glucose (Lonza, Basel, Switzerland) supplemented with 20% foetal bovine serum (Lonza, Basel, Switzerland), 1% penicillin/streptomycin (Lonza, Basel, Switzerland), 1% ultraglutamine (Lonza, Basel, Switzerland), chicken embryo extract (Sera Laboratories International, West Sussex, UK) and bFGF (Sigma-Aldrich, St. Louis, MO, USA) under standard culture conditions (95% humidity, 5% CO_2_, at 37 °C). The medium was changed every other day. To avoid spontaneous myotube formation, the cells were passaged at 70% confluency using 0.25% trypsin and EDTA solution (Lonza, Basel, Switzerland).

### Myoblast characteristics

To confirm the myogenic character of the obtained cells, flow cytometry, immunostaining, and multinuclear tube formation was performed at cell passage 3.

#### Flow cytometry

The purity of the cell population was evaluated using a CD56-antibody-PC5 conjugate (Beckman Coulter, Inc. Brea, CA, USA) by flow cytometer (Beckman Coulter, Inc. Brea, CA, USA). As control IgG1-PC5 was used. As described previously^31^, 0. 25 × 10^6^ cell aliquots were harvested, centrifuged (1200 rpm, 10 min) and resuspended in 100 µl of phosphate buffered saline (PBS), with 2% FBS and 10 µl of an CD56 antibody or isotype control in a 1:200 dilution.

#### Immunofluorescence staining

The presence of myogenic markers was confirmed by immunofluorescence by applying anti-desmin and anti-MHC antibodies (Table 1). The cells were fixed in 4% paraformaldehyde in PBS. They were then washed three times in PBS, followed by a 15 min incubation in 0.1% Triton-X-100 in PBS to permeabilize the cell membranes. The cells were then pre-incubated in 10% goat serum diluted in PBS with 0.1% Triton X-100 (Sigma-Aldrich, St. Louis, MO, USA) to block nonspecific epitopes for an additional 60 min at room temperature. After removal of the blocking serum, the cells were incubated overnight at 4 °C with primary antibody diluted in PBS with 0.1% Triton X-100. The secondary antibody conjugated with fluorochrome was added for 1 h. After three washes in PBS, DAPI (Sigma-Aldrich, St. Louis, MO, USA) was added to visualize the cell nuclei (Sigma-Aldrich, St. Louis, MO, USA). The stained preparations were observed under a Leica DMi8 and an Olympus BX40 fluorescence microscope.

**Table 1:** Antibodies applied for immunofluorescence staining.

Antibody	Manufacturer	Characteristics	Dilution
mouse anti-desmin	Abcam Cambridge (UK)	Myogenic markers	1:200
mouse anti-heavy chain myosin			1:400
rabbit anti-Oct4	Abcam Cambridge (UK)	Nuclear pluripotency markers	1:200
rabbit anti-SOX2			1:250
rabbit anti-c-MYC			1:100
mouse anti-SSEA4			1:200
mouse anti-TRA-1-60			1:100
mouse anti-TNNT2	Abcam Cambridge (UK)	Mature cardiomyocyte markers	1:200
mouse anti-α-MHC			1:200
mouse anti-α-actinin	Sigma-Aldrich St. Louis, USA		1:500
rabbit anti- TUJ1	Abcam Cambridge (UK)	3 germ layers	1:200
rabbit anti-SMA			1:100
mouse anti-AFP			1:200
anti-mouse Alexa Fluor 488	Abcam Cambridge (UK)	Flurochrome conjugated secondary antibody	1:500
anti-rabbit Alexa Fluor 594			1:500
anti-mouse Alexa Fluor 647			1:500
anti-rabbit Alexa Fluor 647			1:500

Oct-4- Octamer-binding transcription factor 4, Sox2-Sex determining region Y-box 2,c-MYC- cellular c-Myc oncogene product, SSEA4- Stage-Specific Embryonic Antigen-4, TRA-1-60- Podocalyxin, TNNT2 – Troponin 2, α-MHC- Myosin Heavy Chain, *α* isoform, TUJ1- Neuron-specific class III beta-tubulin, SMA- Alpha Smooth Muscle Actin, AFP- α-fetoprotein.

#### Multinuclear tube formation

Functional myoblast characteristics, such as multinuclear tube formation, was performed by differentiating the cells in medium consisting of Dulbecco’s modified Eagle’s medium containing 4.5 g/l glucose (Lonza, Basel, Switzerland) supplemented with 2% horse serum (Lonza, Basel, Switzerland), 1% penicillin/streptomycin (Lonza, Basel, Switzerland), and 1% ultraglutamine (Lonza, Basel, Switzerland) under standard culture conditions (95% humidity, 5% CO_2_, at 37 °C) for at least 7 days. The percentage of cells with 2 or more nuclei was assessed by counting in total 500 cells and calculating the polynucleated fraction.

#### Reprogramming of myoblasts to iPSCs

Cells from passage 5 were seeded into 6-well plates at the density of 2 × 10^5^ cells/ml. The next day, cells were transduced with a CytoTune-iPS 2.0 Sendai Reprogramming Kit (Thermo Fisher, Waltham, MA, USA) according to the feeder-free kit protocol. One day after viral transduction, the medium was replaced with fresh standard myoblast medium and changed every other day thereafter. At 7 d post-transduction, the cells were passaged onto Geltrex (Life Technologies, Carlsbad, CA, USA)-coated 6 well plates and cultured in Essential 8 medium (Life Technologies, Carlsbad, CA, USA). The iPSCs were passaged using 50 mM EDTA (ThermoFisher, Waltham, MA, USA) in PBS until the cells showed typical iPSC morphology and tests confirming pluripotency were performed.

### iPSC characteristics

#### Immunofluorescence staining

The respective stainings were performed with the antibodies listed in Table 1 to identify the presence of iPSC pluripotency markers (Sox2, cMyc, SSEA, TRA-1-60). Cells were fixed in 4% paraformaldehyde in PBS. The cells were washed three times in PBS, followed by a 15 min incubation in 0.1% Triton-X-100 in PBS to permeabilize the cell membranes. The cells were then incubated in 10% goat serum diluted in PBS containing 0.1% Triton X-100 (Sigma-Aldrich, St. Louis, MO, USA) to block nonspecific epitopes for an additional 60 min at room temperature. After removal of the blocking serum, the cells were incubated overnight at 4 °C with primary antibody diluted in PBS/0.1% Triton X-100. The secondary antibody, conjugated with fluorochromes, was added for an extra hour of incubation. After three washes with PBS, DAPI (Sigma-Aldrich, St. Louis, MO, USA) was added to visualize the cell nuclei. The stained preparations were observed under a Leica DMi8 and an Olympus BX40 fluorescence microscope.

#### Embryoid body formation

Embryoid bodies (EBs) were generated using the AggreWell™ plate protocol according to the manufacturer’s instructions (Stem Cell Technologies, Vancouver, BC, Canada). Next, the EBs were cultured in so-called suspension culture in low-adhesion Petri dishes. After 5 d, the EBs were seeded onto 15 mm Geltrex-coated cover glasses and cultured as adherent cells for 7d. Subsequently, the EBs were fixed in 4% paraformaldehyde solution as previously described and immunostained for three germ layer derivatives (TUJI, AFP and SMA) with the respective antibodies listed in Table 1.

### Generation and analysis of ACTC- and TNNT2-modified iPSCs

#### Lentiviral Plasmid Construction

Troponin 2 vector: The TNNT2-luc-T2A-Puro-MCMV-GFP was produced by Vector Builder (VectorBuilder Inc., TX, USA). The TNNT2 promoter sequence (116 bp) was cloned as previously described^24^. Firefly luciferase aiming to monitor the cell differentiation, and puromycin resistance selected the desired cells. GFP expression, controlled by an mCMV constitutive promoter, indicated the transduction efficiency and the purity of the cell population (Supplementary Fig. S2A- Lentiviral maps generated by Snap Gene Programme).

ACTC vector: The *hACTC-mcherry-WPRE-EF1-Neo* vector was obtained from System Biosciences (System Biosciences, Palo Alto, CA, USA). This cell differentiation reporter expresses mCherry, enabling visualization of the myogenesis. An additional Neo resistance cassette under the control of the EF1 promoter allows the selection of cells carrying the transgene (Supplementary Figure S2B– map generated by Snap Gene Programme).

Plasmids were amplified in the Stbl3 bacterial strain (Life Technologies, Carlsbad, CA,USA).

#### Lentiviral packaging

Lentiviral particles were packed using a 2^nd^ generation packaging system. Plasmids *TNNT2-luc-T2A-puro-mCMV-GFP* or *hACTC-mcherry-EF1-Neo*, *psPAX* (#12260 Addgene) and *MD2G* (#12259 Addgene) (4:3:1) were mixed, and transfection of the HEK cells (27 × 10^6^) was performed using a calcium phosphate protocol^46^. Pseudoviral particles were collected at 48 and 72 h post transfection, filtered through a 45 µm filter (Millipore, Temecula, CA, USA) and centrifuged using centrifugal filter units (Amicon Ultra-15, Merck, Temecula, CA, USA). The aliquots were snap-frozen and stored at −80 °C.

#### iPSC transduction

One hour before transduction, cells were treated with protamine sulfate (5 µg/ml), followed by a half volume of fresh E8 medium (ThermoFisher Scientific, Carlsbad, CA, USA) supplemented with protamine sulfate (5 µg/ml, Sigma-Aldrich, St. Louis, MO, USA). *TNNT2* and *ACTC* lentiviral particles were added and incubated for 6 h. The virus titre was adjusted experimentally. GFP-positive cells were separated by cloning, whereas selection of cells carrying the ACTC transgene was performed by adding G418 (0, 35 µg/ml, Sigma-Aldrich, St. Louis, MO, USA) to the culture medium. Medium was refreshed every day for up to one week. Immunofluorescent staining and EB formation in the TNNT2/ACTC-modified iPSC line was performed as previously described.

### Insert copy number determination

#### Cell fixation and FISH assay

Cell fixation for FISH analysis was performed according to the protocol described by Moralli *et al*.^47^. Briefly, cells were incubated in 0.4% KCl with HEPES for 30 min at 37 °C and fixed with fixative solution (3:1 methanol/glacial acetic acid).

Probes specific for *TNNT2* plasmid and *ACTC* plasmid were independently labeled with biotin-16-dUTP (11093070910, Roche, Basel, Switzerland) by the random priming method using a commercially available kit (Invitrogen, Carlsbad, CA, USA). A standard protocol for Interphase FISH (I-FISH) experiments was applied. The cells were first treated with RNase A (Roche, Basel, Switzerland) at a concentration of 100 μg/ml for 1 h at 37 °C, washed in 2XSSC buffer and sequentially dehydrated in 70%, 80%, and 90% EtOH. Slide denaturation was then performed by incubating the cells in 70% formamide/2xSSC for 2 min at 72 °C. Next, hybridization buffer (10% dextran sulfate, 2XSSC, 50% formamide, 10% Tween-20) containing 200 ng of biotin-labeled probe was applied to slides, and the cells were incubated under the cover glasses at 37 °C overnight in a humidified chamber. The next day, after gentle washing, the cells were incubated for 30 min at 37 °C in blocking buffer to prevent nonspecific binding (3% BSA, 4XSSC, 0.05% Tween-20). The cells were then incubated with Cy^®^3-streptavidin (GE Healthcare, Chicago, IL, USA) at a 1:200 dilution for 1 h at room temperature. After gentle washing, the nuclei were stained with Vectashield Antifade Mounting Medium with DAPI (H-1200, Vector Laboratories, Burlingame, CA, USA). The cells were evaluated under a Nikon E600 Eclipse fluorescence microscope, and images were taken using Lucia software. Signal counting was performed on 50 nuclei from independent slides and compared with the control (reporter-unmodified iPSCs). The number of analysed cells was chosen in reference to a study published by Qiao *et al*.^48^.

#### Digital droplet PCR (ddPCR)

For each sample, prior to performing the ddPCR reaction, 20 ng of DNA template was digested with the restriction enzyme HindIII (New England Biolabs, Ipswich, MA, USA) for 20 min at room temperature at a final concentration of 5 U/reaction. The total reaction volume was 22 μl and contained digested DNA, 2x ddPCR™ Supermix for Probes (No dUTP) (Bio-Rad Hercules, CA, USA), and primers complementary to luciferase firefly sequences and FAM-labeled probe (luc assay ID dCNS392190807, Bio-Rad Hercules, CA, USA). Each sample included also a reference assay consisted of primers complementary to the AP3B1 gene and HEX-labelled probe (assay ID dHsaCP1000001, Bio-Rad Hercules, CA, USA). The final concentrations of primers and probe in the ddPCR reactions were 900 nM for primers and 250 nM for probe. Droplets were generated using a QX200 droplet generator (Bio-Rad Hercules, CA, USA) according to the manufacturer’s instructions. Droplets were then transferred into 96-well plates and heat-sealed with a PX1 PCR plate sealer (Bio-Rad Hercules, CA, USA). Subsequently, the PCR was run on a Gradient T100 thermal cycler (Bio-Rad Hercules, CA, USA) with the following cycling conditions (with a ramp rate of 2 °C/s): 95 °C for 10 min, followed by 40 cycles of 94 °C for 30 sec (denaturation) and 60 °C for 1 min (annealing/extension), and finally at 98 °C for 10 min. The subsequent reading of the droplets was performed in a QX200 droplet reader (Bio-Rad Hercules, CA, USA). The data obtained was analysed using QuantaSoft software (Bio-Rad Hercules, CA, USA).

#### Quantitative real-time polymerase chain reaction (qPCR)

Genomic DNA was extracted using the Whole Blood Extraction Mini kit (Qiagen, Germantown, MD, USA). The DNA concentration was measured with a NanoDrop. Q-PCR was performed with primers specific for the *TNNT2* plasmid (forward: 5′-CCAGGGATTTCAGTCGATGT-3′ and reverse: 5′-AATCTGACGCAGGCAGTTCT-3′ and for the *ACTC* plasmid (forward: 5′-GGTACAGTGCAGGGGAAAGA-3′, reverse: (5′-CCAGGGCAGATCGATAAAAT-3′). The *TNNT2* and *ACTC* insert copy numbers were normalized to *Albumin* (forward: 5′-GCTTATGGAGGGGTGTTTCA-3′ and reverse 5-’TGGAGACTGGCACACTTGAG-3′). The Q- PCR conditions were as previously published^49^.

### iPSC differentiation into cardiomyocytes

To differentiate *TNNT2/ACTC* reporter-modified and reporter-unmodified iPSCs into cardiomyocytes, a PSC kit was used (Life Technologies, Carlsbad, CA, USA). Modified iPSCs (from passages 34, 37, 40, 43, and 46), as well as reporter-unmodified control cells were seeded into 12-well Geltrex-coated plates. At a confluency of 85%, medium A was added and incubated for 2 d followed by replacement with medium B for another 2 d. Finally, the cells were cultured in cardiomyocyte maintenance medium.

The evaluation of firefly luminescence (referring to *TNNT2* expression) was measured using a luciferase assay system (Promega, Madison, WI, USA) at days 0, 4, 10 and 14. Measurement was performed in triplicate for each indicated day on GloMax luminometer (Promega, Madison, WI, USA). The activity of *ACTC* (mCherry) in differentiating cells was observed under a fluorescence microscope (Leica DMi8) at the same time points.

#### Generation and analysis of TNNT2 and ACTC reporter-containing cardiomyocytes

One hour before transduction, the cardiomyocytes (20 days of cardiac differentiation) were treated with protamine sulfate (5 µg/ml), followed by cardiomyocyte maintenance medium (ThermoFisher Scientific, Carlsbad, CA, USA) supplemented with protamine sulfate (5 µg/ml, Sigma-Aldrich, St. Louis, MO, USA). *TNNT2* and *ACTC* lentiviral particles were added and incubated for 24 h. The virus titre was adjusted experimentally. The evaluation of firefly luminescence was measured at 72 h post transduction (in triplicate) using a luciferase assay system (Promega, Madison, WI, USA). The expression of *ACTC* mCherry was observed under a fluorescence microscope (Leica DMi8).

#### Immunofluorescence staining

Staining of reporter-unmodified and *TNNT2/ACTC* reporter-modified cardiomyocytes was performed with anti-actinin, anti-TNNT2 and anti-cMHC antibodies (listed in Table 1), as previously described in section 2.3. The cells were fixed in 4% paraformaldehyde in PBS. After washing three times in PBS, they were incubated for 15 min in 0.1% Triton-X-100/HPBS to permeabilize the cell membranes. The cells were then incubated for another 60 min at room temperature in 10% goat serum diluted in PBS containing 0.1% Triton X-100 (Sigma-Aldrich, St. Louis, MO, USA) to block nonspecific epitopes. After removal of the blocking serum, the cells were incubated overnight at 4 °C with primary antibody diluted in PBS with 0.1% Triton X-100. The secondary antibody conjugated with fluorochrome was added and incubated for 1 h. After three washes with PBS, DAPI (Sigma-Aldrich, St. Louis, MO, USA) was added to visualize the cell nuclei (Sigma-Aldrich, St. Louis, MO, USA). The stained preparations were observed under a Leica DMi8 and an Olympus BX40 fluorescence microscope under 100x magnification.

### Statistical analysis

The statistical analysis was performed using Graph Pad Prism software, version 5.03 for Windows. The data are presented as the mean ± SD, *p < 0.05, **p < 0.01, ***p < 0.001.

## Supplementary information

Supplementary movie.

Supplementary information.
