# Supplementary material for: Tissue-specific promoter-based reporter system for monitoring cell differentiation from iPSCs to cardiomyocytes
Source: Sci Rep. 2020 Feb 5;10:1895. doi: 10.1038/s41598-020-58050-2 (PMC7002699; doi:10.1038/s41598-020-58050-2)
Supplement: Supplementary file 2 — Supplementary information. [file 41598_2020_58050_MOESM2_ESM.pdf]

Supplementary material

Tissue-specific promoter-based reporter system for monitoring cell differentiation from iPSCs to cardiomyocytes

Katarzyna Fiedorowicz[1*], Natalia Rozwadowska[1*], Agnieszka Zimna[1], Agnieszka Malcher[1], Katarzyna Tutak[1], Izabela Szczerbal[2], Karolina Nowicka-Bauer[1], Magdalena Nowaczyk[1], Tomasz J. Kolanowski [1], Wojciech Łabędź[3], Łukasz Kubaszewski[3], Maciej Kurpisz[1]

*Authors equally contributed to the study

[1]Institute of Human Genetics, Polish Academy of Sciences, Poznan, Poland,

[2]Department of Genetics and Animal Breeding, Poznan University of Life Sciences, Poznan, Poland,

[3]Department of Spondyloortopaedics and Biomechanics of the Spine, W. Dega University Hospital, Poznan University of Medical Sciences, Poznan, Poland

Corresponding author:

Maciej Kurpisz MD, PhD

Institute of Human Genetics, Polish Academy of Sciences

Strzeszynska 32

60-479 Poznan, Poland

e-mail: maciej.kurpisz@igcz.poznan.pl

phone: +(48) 61 6579202

Supplementary Figure S 1. Determination of ACTC copy number in TNNT2/ACTC reporter-modified iPSCs.

A. Representative image of I-FISH signals (15 per cell) with complementary probe generated from ACTC vector for TNNT2/ACTC-modified iPSCs at passage 40. Unmodified cells were used as negative control. Images were taken at 100x magnification.

B. The average ACTC copy number in cell populations was estimated in cells at passages 34, 37, 40, 43, and 46 and unmodified cells by qPCR; values were given as the mean ± SD for qPCR triplicates; *p < 0.05, **p < 0.01, ***p < 0.001.

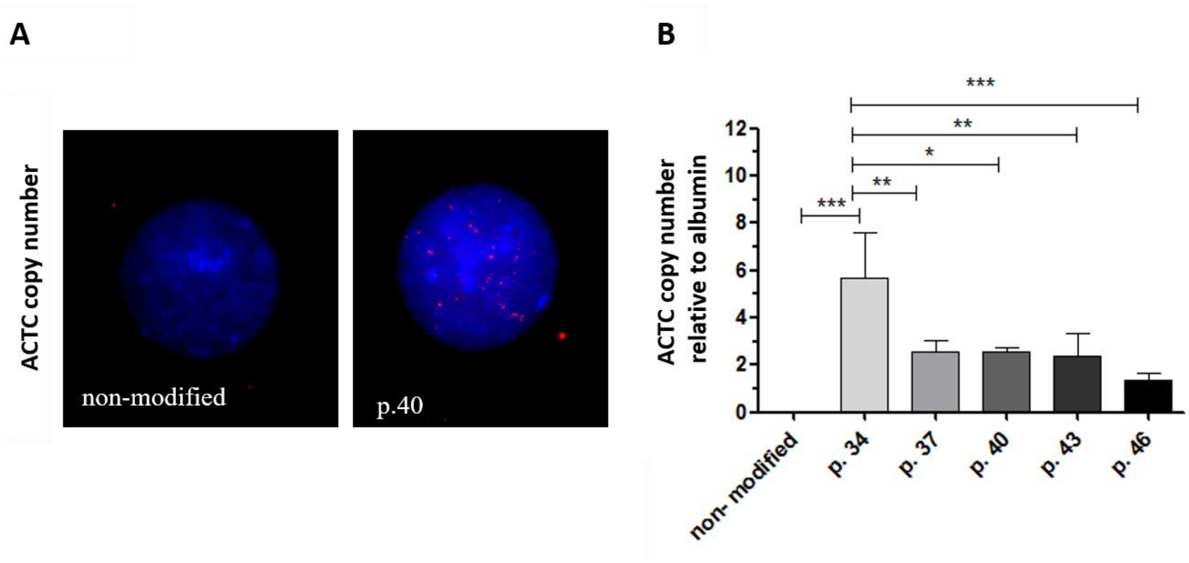

Supplementary Figure S 2. Lentivirual maps generated by Snap Gene Programme.

A. *TNNT2-luc-T2A-Puro-mCMV-GFP*: Firefly luciferase monitors differentiation into cardiomyocytes, and puromycin resistance selects target cells. GFP expression controlled by a mCMV constitutive promoter illustrates the transduction efficiency.

B. hACTC-mCherry-WPRE-EF1-Neo: This vector expresses mCherry, which enables visualization of myogenesis. The neo resistance cassette under the control of the EF1 promoter allows for selecting cells carrying the transgene.

A

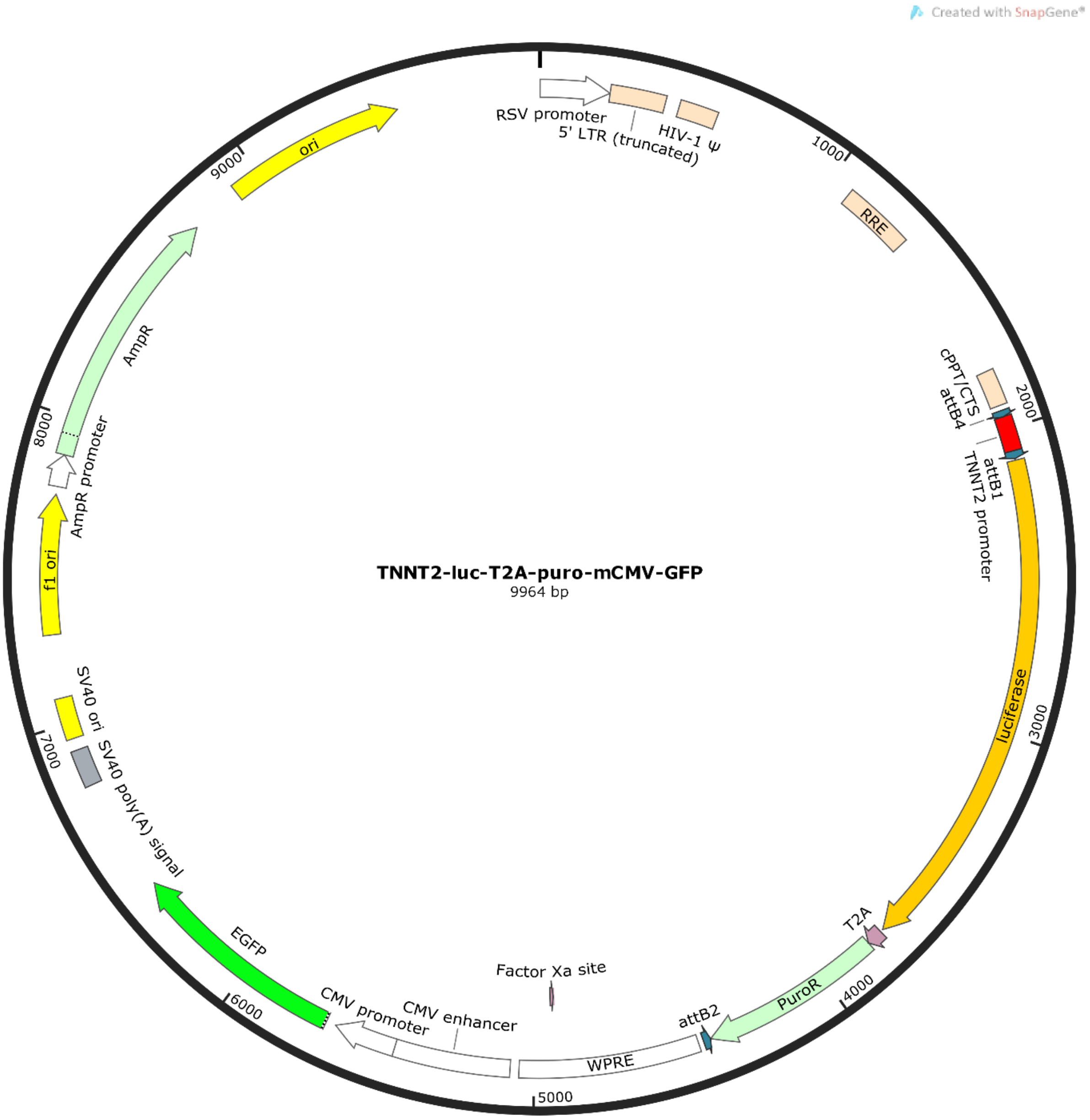

B

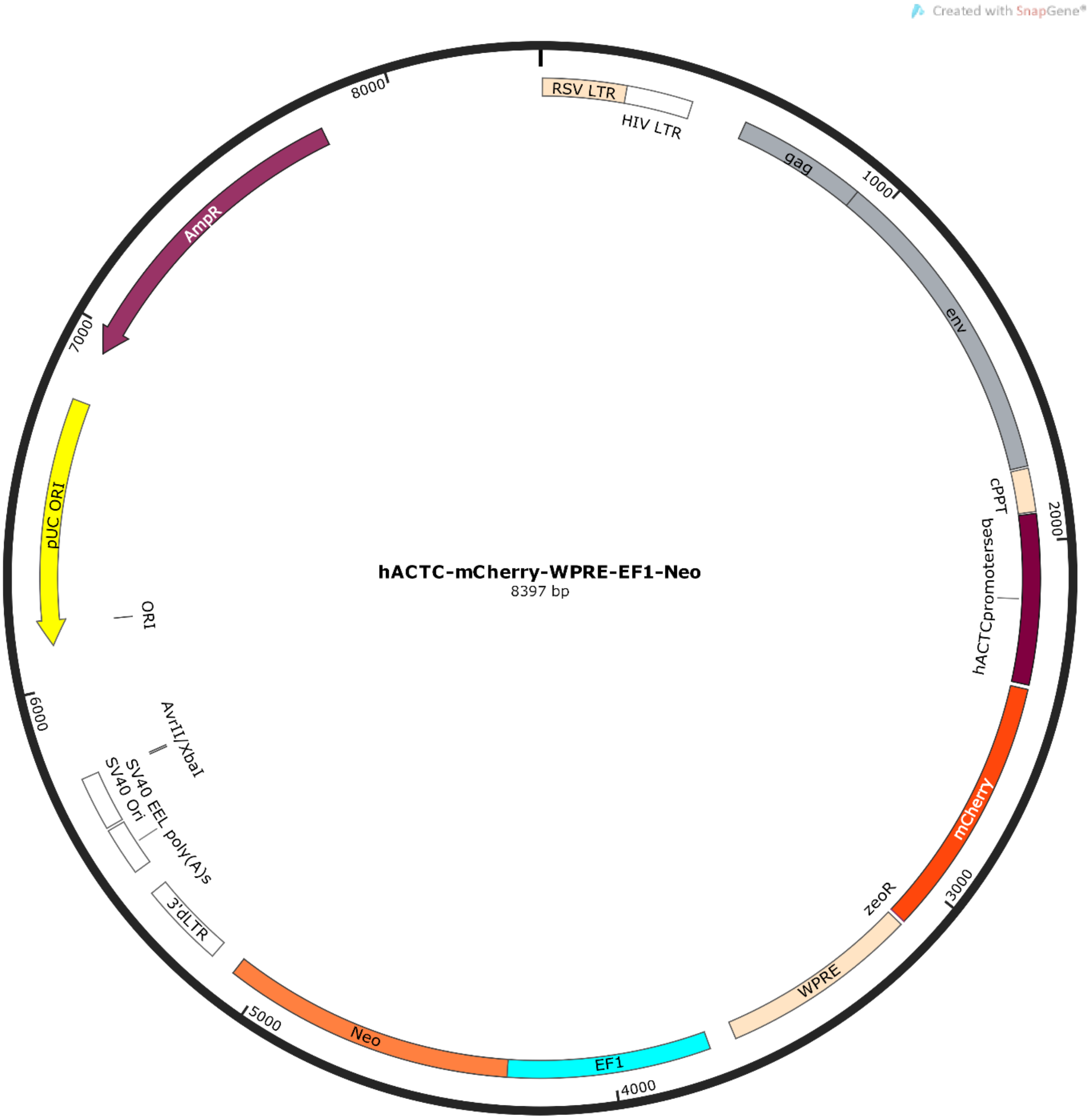

Created with SnapGene®
hACTC-mCherry-WPRE-EF1-Neo
8397 bp
RSV LTR
HIV LTR
gag
1000
env
cPPT
2000
hACTCpromoterseq
mCherry
3000
zeoR
WPRE
EF1
4000
Neo
5000
3'dLTR
SV40 Ori
SV40 EEL poly(A)s
AvrII/XbaI
6000
ORI
pUC ORI
7000
AmpR
8000

Movie 1 TNNT2/ACTC reporter-modified cardiomyocytes
